# Novel EM Guided Endovascular Instrumentation for In Situ Endograft Fenestration

**DOI:** 10.1109/JTEHM.2020.2973973

**Published:** 2020-03-04

**Authors:** S. Condino, R. Piazza, R. M. Viglialoro, D. M. Mocellin, G. Turini, R. N. Berchiolli, F. Micheletti, F. Rossi, R. Pini, V. Ferrari, M. Ferrari

**Affiliations:** 1 Information Engineering DepartmentUniversity of Pisa 9310 56122PisaItaly; 2 EndoCAS CenterDepartment of Translational Research and New Technologies in Medicine and SurgeryUniversity of Pisa 9310 56126PisaItaly; 3 Vascular Surgery UnitCisanello University Hospital AOUP56126PisaItaly; 4 Computer Science DepartmentKettering University 3364 FlintMI48504USA; 5 Institute of Applied Physics “Nello Carrara,” National Research Council50019Sesto FiorentinoItaly

**Keywords:** Electromagnetic guidance, endovascular instrumentation, EVAR, laser fenestration, in situ fenestration

## Abstract

*Objective:* This work aims at providing novel endovascular instrumentation to overcome current technical limitations of in situ endograft fenestration including challenges in targeting the fenestration site under fluoroscopic control and supplying mechanical support during endograft perforation. *Technology:* Novel electromagnetically trackable instruments were developed to facilitate the navigation of the fenestration device and its stabilization at the target site. In vitro trials were performed to preliminary evaluate the proposed instrumentation for the antegrade in situ fenestration of an aortic endograft, using a laser guidewire designed ad hoc and the sharp end of a commercial endovascular guidewire. *Results:* In situ fenestration was successfully performed in 22 trials. A total of two laser tools were employed since an over bending of laser guidewire tip, due to its manufacturing, caused the damage of the sensor in the first device used. *Conclusions:* Preliminary in vitro trials demonstrate the feasibility of the proposed instrumentation which could widespread the procedure for in situ fenestration. The results obtained should be validated performing animal studies. Clinical Impact: The proposed instrumentation has the potential to expand indications for standard endovascular aneurysm repair to cases of acute syndromes.

## Graphical abstract

Overview of in vitro setup for laser in situ fenestration (a): the electromagnetic navigation system scene (b) allows the user to position the novel laser endovascular instrumentation at the target blood vessel origin (visible also in the endoscopic view). A successful laser in situ fenestration is shown in the real scene (c), where the sensorized laser guidewire perforated the endograft wall at left renal level.

## I. Introduction

Endovascular aortic aneurysm repair (EVAR) is a popular, minimally-invasive surgical technique for treating abdominal aortic aneurysms (AAAs). Real-time bi-dimensional X-ray fluoroscopic images are acquired by using a C-arm, a medical imaging device allowing the surgeon to monitor progress and navigate endovascular instruments during the intervention. Then, an endograft (a covered stent) is deployed to exclude the aneurysm from the blood flow, preventing its expansion and rupture.

Potential advantages of EVAR over traditional open repair include: reduction of the time under general anesthesia, less pain and trauma and blood loss, and shorter hospitalization [1]. Moreover, the low 30-day mortality and morbidity of this endovascular technique renders it particularly appropriate for the treatment of high-risk surgical patients [2]. However, as documented in recent literature [3], 30–40% of AAAs cannot be treated with traditional EVAR due to issues such as short infrarenal neck (<10 mm), and inclusion of visceral vessels.

To preserve the perfusion of aorta branches (e.g. renal and middle suprarenal arteries, and visceral arteries) is a critical aspect in EVAR procedures, and it drastically influences the outcome of these surgical interventions. Technical advances in fenestrated graft and branched endograft technologies have enabled endovascular repair of complex aneurysms, previously considered ineligible. Fenestrated endovascular aneurysm repair (FEVAR) allows the perfusion of renal and visceral arteries through holes, i.e. fenestrations, in the graft. These fenestrations must be accurately aligned with the ostia of target vessels, and this requires high technical skill and precision. Moreover, this procedure requires several readjustments of the C-arm pose (to optimize the viewing direction) which result in prolonged radiation exposure [2]. Finally, fenestrated grafts are customized to fit the patient anatomy, so their fabrication is expensive and time-consuming (tailored process can exceed 6 weeks [4]), and therefore they cannot be used for the emergency treatment of acute syndromes.

For these reasons McWilliams *et al.* [5], [6] in 2003 proposed an interesting alternative, the in situ fenestration (ISF), an intraoperative technique capable of avoiding the long waiting times for the fabrication of a fenestrated graft. The ISF consists in creating holes in a standard endograft, immediately available for the patient, which is inserted within an aneurysm, and secured to the aortic wall, thus occluding branched blood vessel origins (ostia). The fenestration must be performed within a safe interval time to avoid renal ischemia. Since the ISF original paper, several reports have been published describing the retrograde ISF, which implies downstream blood vessel access to fenestrate the endograft from the outside (typically for restoring blood flow at aortic arch level) [7]. This retrograde technique, however, cannot be adopted to create holes in an abdominal endoprosthesis since it would entail a surgical approach to the target branched vessels, involving large incisions and deep dissections. Instead, in the abdominal aorta, ISF requires the antegrade approach, which allows the modification of the endograft fabric from inside. As emerge from a recent review [7], the antegrade procedure is much more complicated than the retrograde operation and some technical issues have still to be overcome:

• difficulties in identifying the proper fenestration site;
• lack of endovascular devices able to provide the necessary support to perform ISF precisely.

Other major requirements are:

• selective fenestration of the graft material without risk of damaging the arterial wall;
• precise enlargement of the initial hole to guarantee mechanical integrity and fatigue resistance of fenestrated fabric;
• long-term stability of the fenestrated component.

This work proposes a solution to the first two issues mentioned above: it describes a method to navigate the fenestration instrument to the target site; and presents for the first time, innovative endovascular catheters specifically designed to provide the required support during an ISF procedure. In particular, this paper reports the detailed description of the hardware components and usage guidelines for the proposed instrumentation, designed to be used with a custom endovascular navigator. Moreover, results of in vitro evaluation of ISF procedures are presented. Finally, potential clinical impact, limitations, and possible solutions of the proposed technology are illustrated.

## II. Background and Approach

Proper identification of the target fenestration site is one of the main issues of the antegrade ISF: after the endograft deployment, the flow of the contrast agent is blocked by the endograft wall, hindering the view of aorta branches by angiographic imaging.

Our research group and others [8]–[11] have demonstrated that a 3D electromagnetic (EM) navigation system, allowing the real-time tracking of endovascular instrumentation, is a trustworthy solution to guide endovascular procedures. Our previous studies provide: a C-arm calibration method to coherently merge the patient virtual model and EM data [8], an estimation of the targeting accuracy of the catheter navigation in static conditions (1.2 ± 0.3 mm) [8], and an evidence that simultaneous EM navigation of guidewires and catheters is feasible without the use of live fluoroscopic images, preserving user performance in term of execution time (e.g. in our in vitro study a group of experienced surgeons took on average about 1.3 minutes to cannulate a renal artery either with fluoroscopy and with EM navigation alone) [9].

The main idea of this work is to provide the surgeon with EM guided endovascular instruments designed so that their 3D pose (i.e. position and orientation) can be accurately tracked in real-time. This allows the visualization of virtual tool replicas within a 3D model of the patient vasculature. Such anatomical model can be generated by the segmentation of an intraoperative 3D dataset (e.g. with a calibrated rotational C-arm) acquired before the endograft deployment for a complete 3D reconstruction of the target aorta branches. Thus, this method allows the visualization of the aorta branches before and after endograft deployment, since it does not rely on the use of fluoroscopy and contrast agent. Miniaturized EM coils can be used for the real-time tracking of the guidewire and the catheter, and to reconstruct the distal curvature of the latter, as in [9].

For the specific ISF procedure, the existing EM navigation platform [12] is equipped with trackable catheters designed ad hoc, which can be used in conjunction with different fenestration tools.

Reported methods for endograft fenestration prevalently use commercially available endovascular instruments beyond their usual designations [7], for example: laser atherectomy catheters designed for atherosclerotic plaque vaporization [13]–[15], wires [6] and hollow needles [16], [17]. According to [7], the only devices specifically designed for ISF are balloon-centered and balloon-anchored needle-dilator catheters and radio frequency (RF) plasma electrode catheters designed to treat the aortic arch [18].

In [19], [20] we have proposed the development of a custom-built sensorized laser tool emitting in the near-infrared wavelength: a novel instrument for a rapid, repeatable, and selective strategy for ISF. In an in vitro study conducted on 225 of human aorta samples [21], [22], we have demonstrated that the proposed fenestration tool is harmless, since it causes negligible or no injuries to the biological tissue, even in the presence of unintentional direct irradiation of the aorta wall (due to an inaccurate targeting of the fenestration site).

## III. Methods and Procedures

This section provides details on the architecture of our 3D EM navigation system and describes both the design and usage of the innovative endovascular instrumentation proposed. Moreover, the methods employed to preliminary test the EM-guided ISF procedure are reported, together with the evaluation results.

### A. Endovascular Navigator Architecture

**FIGURE 1.**
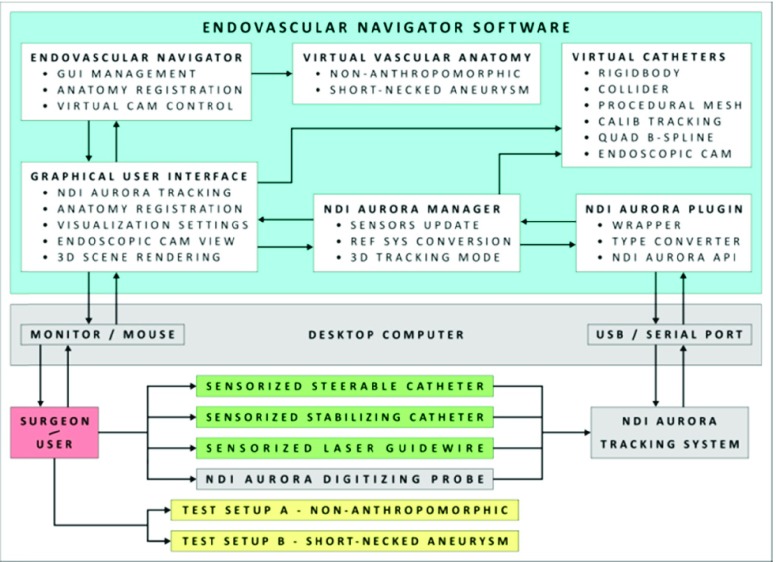
Diagram of the endovascular navigator: the software (in cyan) and the hardware (in grey) modules, the surgeon/user (in red), the developed instrumentation (in green), testing in vitro setups (in yellow). Physical interactions and data transfers are represented with directional lines.

The basic architecture of the proposed EM guided endovascular navigation system is reported in Fig. 1.

The EM navigation software, developed using the Unity game engine, consists of modules/scripts to manage:

• the graphical user interface (GUI),
• the real-time communication with the NDI Aurora EM tracking system,
• the registration-calibration procedure to align the image coordinate system to the coordinate system of the Aurora device, and
• the real-time visualization of the endovascular instrumentation inside the 3D patient-specific vasculature model.

The transmission of the NDI Aurora data to the EM navigation software is managed through a custom Unity Plugin, the NDI Aurora Unity Plugin, which includes: a module (C ++ DLL) for the NDI Aurora API; a module to perform type conversion from C ++ (NDI Aurora API) to C# (Unity); and a module wrapping the NDI Aurora API functions used in Unity (C# scripts) [12].

The NDI Aurora Unity Plugin is used by the NDI Aurora Manager Script, which: acquires EM data at a constant update rate (40 Hz); manages the conversion from the Aurora reference system to the Unity 3D space; and updates in in-real time the 3D pose of the virtual tools accordingly to the acquired EM data properly calibrated (depending on the number and the relative positioning of the EM coils in the tool, as detailed in [9]).

The virtual catheters are implemented using a procedural 3D model updated in real-time accordingly to the configuration of a structure of colliders, rigid bodies, and joints: arranged using a quadratic B-spline derived in real-time from the calibrated 3D pose of the EM sensors embedded in the catheter shafts. RigidBody, ConfigurableJoint, and CapsuleCollider components are used to model each segment of the virtual catheter, and the Unity Physics Engine enables the interaction (collision detection and physics simulation) of the virtual catheter with the 3D anatomical model. Additionally, a Camera component is positioned on the tip catheter model to provide the surgeon/user with an endoscopic view. The mesh of the anatomy can be registered thanks to the Anatomy Registration Module, which implements a least-squares fitting of two 3D point sets [23] to calculate the rigid static transformation between the 3D C-arm and the Aurora reference system, using a simple calibration phantom [8]. The software frame rate is ~35 fps.

### B. Trackable Laser Tool

**FIGURE 2.**
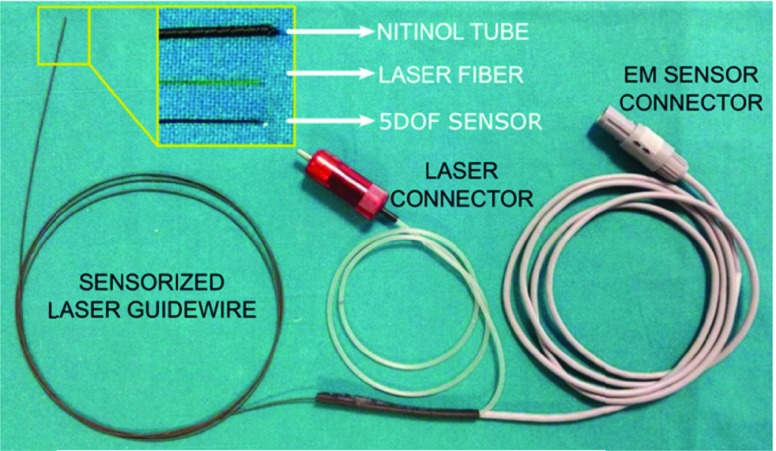
Overview of developed sensorized laser guidewire. The EM coil and the laser fiber are both integrated within the nitinol hollow strand, as shown in the zoomed detail.

A sensorized laser guidewire was developed to guarantee the selective fenestration of the endograft. Nowadays, the optical fibers used in laser therapy are silica fibers surrounded by a protective coaxial coating. In this work, a silica optical laser fiber was integrated into a guidewire, together with a 5DOF (5 degrees of freedom) EM sensor coil (0.3 mm in diameter and 13 mm in length). This enabled an atraumatic advancement of the guidewire through the vascular system, monitored in real-time by the user thanks to the NDI Aurora EM tracking system. More in particular, the prototype of the sensorized laser guidewire consists of a 0.22 Numerical Aperture (NA), high-power multimode fiber ($200~\mu \text{m}$ in diameter) (Thorlabs Inc., Newton, NJ, USA), inserted into a nitinol helical hollow strand (0.035 in. in diameter and 180 cm in length) (Fort Wayne Metals, Fort Wayne, IN, USA), suitable for endovascular applications. The EM sensor coil is secured close to the nitinol tube tip (Fig. 2).

The selected optical fiber has a double-clad construction (TECS hard coating over fluoride-doped silica cladding) and exhibits bending performances adequate for the target procedure since its short-term bend radius (i.e. the minimum radius allowed during use) is 12 mm.

### C. Trackable EM Catheters

The proposed instrumentation consists of two sensorized EM catheters designed ad hoc to: accurately guide the fenestration tool without causing injury to the arterial wall, properly orient the fenestration tool towards the target fenestration site, and offer mechanical support and stability during fenestration.

More specifically: a 9 Fr steerable catheter (with a working length of 800 mm), was designed to properly position and orient the fenestration tool toward the fenestration site; and a 15.24 Fr stabilizing catheter (with a working length of 700 mm), hereafter referred to as the “stabilizer”, was designed to offer mechanical support.

**FIGURE 3.**
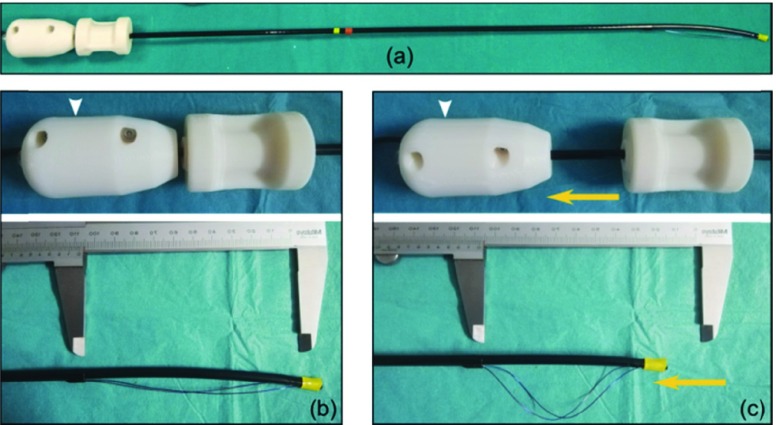
Overview of stabilizer (a): from navigation configuration (b) the nitinol components are expanded in working configuration (c) by pulling (yellow direction) the slidable handle (white arrowhead).

The stabilizer has nitinol expandable components, namely two nitinol wires that can be operated through a slidable handle (Fig. 3). The stabilizer consists of two coaxial tubular structures, respectively connected to the proximal and distal ends of the nitinol wires. The coaxial movement of the two tubes, operated by the catheter handle, allows the stabilizer to switch between two different configurations:

• the navigation configuration, with nitinol components retracted to facilitate the piloting of the catheter inside the patient vasculature (Fig. 3b);
• the working configuration, with nitinol components fully expanded to push the catheter against the endograft wall and stabilize it in front of the fenestration target (Fig. 3c).

**FIGURE 4.**
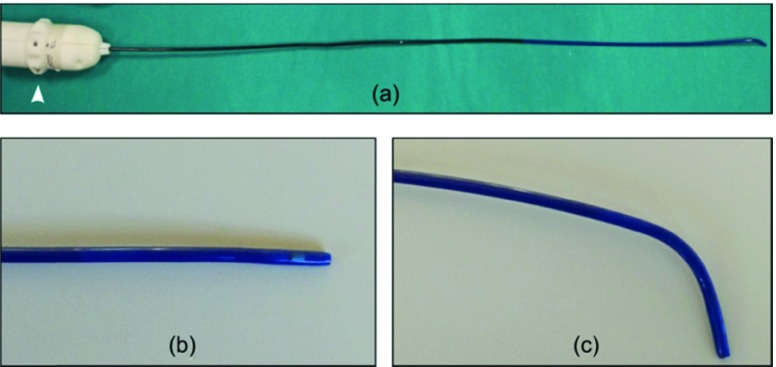
Overview of steerable catheter (a): the straight distal part of the tip (b) can be bent (c) by turning the cog (white arrowhead) of the handle.

The steerable catheter can be easily rotated to appropriately orient the fenestration tool in the direction of the target site. To perform a perfect fenestration, this tool and the steerable catheter tip, should be perpendicular to the graft surface [24], [25]. For this reason, the catheter was designed to allow for a 90° articulation. More specifically: the catheter has 1DOF of bending (articulation in a single plane) actuated via a cable system, manually controlled via the catheter handle (Fig. 4).

In order to prevent any damage to the laser fiber due to over-bending, the design of the steerable catheter distal end has taken into account the short-term bend radius of the optical fiber used. In addition, the two sensor coils, one used for the fenestration tool and another inserted in the catheter tip, should not be bent during the orientation at the target site. Thus, the steerable catheter tip, with a length of 13 mm, does not bend.

Both the stabilizer and the steerable catheter feature a multi-lumen, braided structure made of 304V stainless steel, an austenitic non-ferromagnetic steel, chosen to avoid EM interference with the NDI Aurora tracking device. The internal structure of the metal allows the catheter to meet torqueability and pushability specifications [24]. The torqueability is the capability of a catheter to transmit twisting forces without kinking allowing the rotation of its distal end by turning its proximal portion; whereas the pushability is the capability of a catheter to transmit the force along its main axis for an easy advancement into the vascular system.

Two 5DOF EM sensors (0.5 mm in diameter and 8 mm in length) are employed to track the catheters. The accuracy, measured as root mean square error (RMSE), of these EM coils used in combination with the NDI Aurora Planar Field Generator, is 0.70 mm for translation and 0.20° for rotation in an environment free of EM disturbances.

Thanks to a calibration procedure [19], these EM coils enable: to calculate in real-time the position of the catheter tip, to derive the catheter tip axis and the second sensor axis, and to estimate any deformation of the catheter distal part (the catheter section between the two sensors).

Both catheters were prototyped by Creganna Medical (Galway, Ireland), a company specialized in endovascular catheter design and manufacturing.

### D. In Vitro Testing Environments and Study Protocols

**FIGURE 5.**
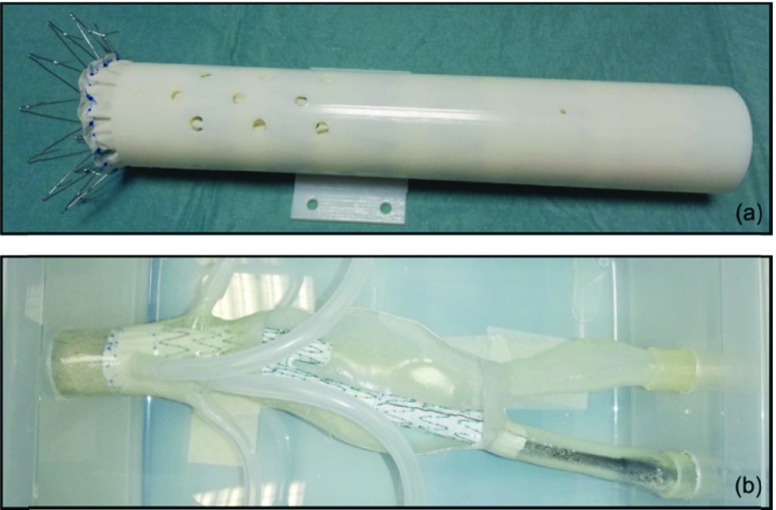
In vitro testing setups: Setup A, a non-anthropomorphic phantom (a); and Setup B, a phantom of a short-necked aneurysm (b).

Two in vitro environments (Fig. 5), with different levels of complexity, were developed to test the proposed EM instrumentation.

The first testing environment, Setup A, is a non-anthropomorphic phantom with multiple fenestration targets (Fig. 5a), that was used for an extensive technical evaluation of the EM catheter system to guide and stabilize the fenestration tool. This system was tested with both the custom laser tool and in conjunction with a simpler alternative mechanical fenestration tool: the sharp-end of a commercial endovascular guidewire.

The second in vitro setup, Setup B, is a synthetic model of a short-necked aneurysm (Fig. 5b); it was manufactured for the preliminary end-user testing of the proposed navigation platform, and to perform a laser ISF in correspondence to the origin of renal arteries.

***Setup A – Multiple Mechanical and Laser ISF Tests***

Setup A consists of a Zenith TX2 thoracic endograft (Cook Medical, Bloomington, IN, USA) inserted into a simple tubular structure, designed ad hoc with a series of holes (Fig. 5a) to simulate the ostia of the arteries originating from the abdominal aorta. The diameters of the holes (5 mm or 4 mm) were selected to be smaller than those of the aorta branches (mean literature values [26]: celiac artery 6.4 mm, superior mesenteric artery 7.3 mm, right renal artery 5.23 mm, left renal arteries 5.1 mm). Moreover, three additional holes (2 mm) were designed in known positions and used for a point-based registration with least squares error minimization algorithm [23].

To assess the system functionalities the following repetitive tests were carried out: 10 mechanical fenestration trials, and 10 laser fenestration trials with targets of different sizes (for both trials, half of the targets had a diameter of 4 mm and the other half a diameter of 5 mm) and positions/orientations.

As already mentioned, mechanical ISF tests were performed by using a non-sensorized tool: sharp-end of a 0.014 in Hi-Torque Whisper guidewire (Abbott Vascular Co., Santa Clara, CA, USA).

Laser ISF tests were performed connecting the sensorized laser guidewire to a diode laser (SMARTY A800; DEKA, Calenzano (FI), Italy) emitting at 810 nm, with a maximum power output of 10 W. The following operative conditions (harmless both for the sensor [19] and for the biological tissue [21]) were set up: irradiation time 500 ms, and irradiation power 4.0 W.

All fenestration trials were performed by a single technical operator, skilled in the use of 3D EM navigation systems (more than 5 years of experience). Each fenestration trial, performed under the guidance of the 3D navigation system, included the following steps:

1) Introduction of the stabilizer.
2) Longitudinal advancement of the stabilizer in the main body of the endograft up to the fenestration target.
3) Adjustment of the stabilizer orientation, and opening of the stabilizing components.
4) Introduction of the steerable catheter into the stabilizer lumen, until its steerable distal end emerges beyond the tip of the latter.
5) Introduction of the fenestration tool (sharp-edged guidewire or laser guidewire), up to the steerable portion of the catheter.
6) Steering of the catheter tip.
7) Final adjustment of the steerable catheter position and orientation (and the stabilizer if necessary), and targeting of the desired fenestration site with the steerable catheter tip.
8) Advancement of the fenestration tool beyond the tip of the steerable catheter.
9) Fenestration of the endograft via a mechanical puncture or laser irradiation.
10) Advancement of the fenestration tool through the newly opening for at least 2 cm.

During the laser fenestration, the user pushed the laser guidewire against the wall of the endograft to help it pass through the newly created hole.

***Setup B – Laser ISF End-User Testing***

The vascular 3D model used in Setup B was manufactured starting from the acquisition of a real computed tomography (CT) dataset using contrast agent. The EndoCAS Segmentation Pipeline [27], a semi-automatic segmentation tool integrated into the open-source software ITK-SNAP, was used to process the generated DICOM (Digital Imaging and COmmunications in Medicine) dataset. Then, artifacts removal and mesh smoothing stages were performed [28] to optimize the vascular 3D model. Finally, a 3D printer, Objet30Prime (Stratasys, Los Angeles, CA, USA), was used to fabricate a transparent tangible 3D synthetic vascular model (Fig. 5b).

A Zenith Alpha abdominal endograft bifurcated main body (Cook Medical, Bloomington, IN, USA) was manually inserted into the aneurysm, covering both renal arteries: the endograft insertion was made through a dedicated aperture, designed on the aneurysm wall. The vascular model was covered with a surgical drape to prevent direct vision of the instrument navigation in this synthetic environment.

The laser ISF tests were performed with the same operative irradiation conditions used for the tests carried out using Setup A.

A vascular surgeon was recruited to participate in the study. Before starting the tests, he completed a learning session on the navigator functionalities and the custom sensorized surgical tools, followed by a training session on a generic aneurysm phantom (different from the setup used in the tests) to familiarize with the sensorized devices [29].

The trials started with the insertion of the stabilizer via a 20 Fr sheath. The stabilizer was navigated to the endograft main body and then, once the proper longitudinal alignment was reached, the stabilizing components were partially opened. Subsequently, the steerable catheter was advanced through the stabilizer and oriented towards the left renal ostium. The position and the orientation of the stabilizer and the steerable catheter were adjusted, and then the stabilizing components were fully opened. Completed the stabilization, the endograft was fenestrated using the sensorized laser guidewire. Finally, after the fenestration was finalized, the guidewire was advanced through the newly created fabric hole into the renal artery. Afterward, the contralateral side (right renal ostium) was fenestrated using the same methodology.

Passive support was provided by an assistant (e.g. to interact with the navigator GUI and properly orient the 3D vascular model according to the surgical step) during the navigation procedure and the ISF, but hands-on assistance was not allowed.

## IV. Results

Results of the fenestration tests are summarized in Table 1.

**TABLE 1:** ISF Success Rate

	Setup A	Setup B
Mechanical ISF		
Successful fenestration	10/10	No trial
Successful advancement	10/10	performed
Laser ISF		
Successful fenestration	10/10	2/2
Successful advancement	5/10	2/2

***Setup A – Multiple Mechanical and Laser ISF Tests***

ISF was successfully performed in all cases (20/20 targets) at the first attempt.

**FIGURE 6.**
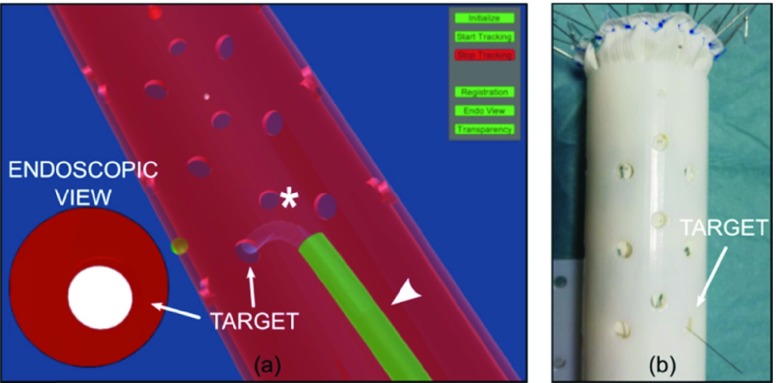
EM-guided mechanical ISF: the navigator scene (a) allows the user to position the stabilizer (white arrowhead) and orient the steerable catheter (asterisk) at the target site (visible also in the endoscopic view). A successful mechanical ISF is shown in the real scene (b), where the sharp-end of the guidewire perforated the endograft wall.

After mechanical fenestration, the sharped-end of the commercial guidewire was easily advanced through the hole, as shown in Fig. 6.

During laser ISF the user noticed some difficulties in pushing the laser tool through the newly created hole. Indeed, after successful fenestrations, the sensorized laser penetrated in the graft for few millimeters until it was jammed in five trials. At this point, it was observed that the helical strand construction of custom guidewire caused the indentation of its tip against the graft wall when a force was applied to advance the tool through the fenestration. A sensor was damaged during a laser ISF test due to the EM coil over-bending related to the indentation issue. This issue required the substitution of the laser tool: a total of two laser tools were used for ten fenestrations. During ISF, some carbonized endograft material deposited on the fiber; however, the same laser guidewire was successfully used for five trials, without requiring any variation of laser irradiation conditions.

***Setup B – Laser ISF End-User Testing***

**FIGURE 7.**
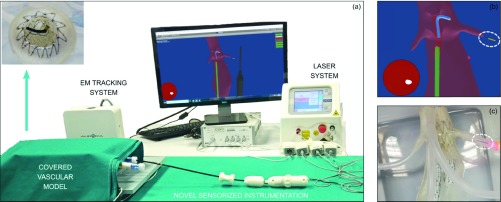
Overview of Setup B for laser ISF (a): the navigator scene (b) allows the user to position the stabilizer (green) and orient the steerable catheter (blue) at the target site (visible also in the endoscopic view). A successful laser ISF is shown in the real scene (c), where the sensorized laser guidewire perforated the endograft wall at left renal level.

The user successfully performed ISF at both renal arteries levels. The cannulation of the right renal artery was obtained at the first attempt. On the contrary, five attempts were necessary to advance the laser fiber through the newly created hole into the left renal artery (Fig.7), due to the aforementioned difficulties in pushing the laser fiber through the newly created hole.

## V. Discussion

The preliminary results obtained demonstrate the feasibility of antegrade ISF using the proposed EM trackable endovascular instrumentation, which allows the surgeon to navigate the fenestration tool to the target surgical site, also offering support during the ISF procedure.

A major limitation of the study is that in vitro tests were performed in a simplified rigid environment, and the anthropomorphic simulator was not provided with a system for the reproduction of the physiologic movements of renal arteries and blood flow simulation. In a real clinical scenario, targeting the ostia would be complicated by the deformations of the aorta due to the endograft delivery, and cardiac cycle and respiration. For example: the right renal ostia moves 1.2 mm and 0.2 mm in the superior-inferior (SI) and antero-posterior (AP) directions respectively, and the left ostia moves 1.6 mm and 0.3 mm in the SI and AP directions, respectively [30].

A robust strategy to guarantee an accurate targeting, even in the presence of ostia movements and aorta deformations, could be based on the development of micro radiopaque trackable guidewires ($\mu $EMg), incorporating multiple miniaturized EM coils, to be deployed in renal arteries before delivering the endograft. These guidewires could be used to monitor in real-time the target ostia displacement and coherently update the 3D anatomical model of the navigator. This can greatly improve the accuracy of the navigator, as the relative positions of the ostia and the sensorized catheter tip are determined in real-time based on EM data, correcting any misalignment of the virtual anatomical model due to inaccuracies in the calibration of the imaging source (3D C-arm), and possible movements/displacements of the vascular anatomy during the surgical procedure. These are the major error sources in image-based navigation systems. The final accuracy will be mainly affected by three sources of error: the EM tracking, which may be adversely affected by interference arising from the environment; the calibration of the sensorized instruments; the reconstruction of the 3D poses of aorta branches from EM coils embedded in the $\mu $EMg. The employed localization system, as well as the EM tools calibration procedure, have a sub-millimeter accuracy [8]. The accuracy in reconstructing the aorta branches poses from $\mu $EMG data is expected to be lower than 1.5 mm, according to our previous experience in using multiple EM coils, inserted into nitinol helical hollow strand, to track tubular structures [31]. The final accuracy can be improved by using: shielded and isolated sensor coils to reduce any EM interference; a self-centering mechanism incorporated into $\mu $EMG tip (e.g. a micro-balloon) to guarantee an accurate alignment of these landmark structures to the target vessel centerline.

From a mechanical design point of view, the proposed trackable catheters showed good results in terms of navigation and mechanic support offered during ISF. However, the difficulties experienced during laser trials highlighted the need to improve the pushability of the laser tool (i.e. increase the rigidity of the nitinol helical hollow strand) to ease its advancement through the newly created hole. Moreover, different solutions will be studied to optimize the safety of the instrumentation, to enlarge the laser spot size, and to ease the advancement into the fenestration: a solution could be based on the use of a ball lens fiber tip, sealed to the nitinol helical strands.

## VI. Conclusion

ISF is an intraoperative technique developed to avoid the intrinsic delays in the fenestrated endograft manufacturing process (nowadays their lead-time exceeds more than 6 weeks [4]) and to offer a bailout alternative in case of side branch occlusions. This technique was originally described as a retrograde intraoperative modification of a standard endograft at the arch level.

This paper presents an innovative EM trackable endovascular instrumentation, designed ad hoc to accurately and safely perform ISF in the abdominal aorta, overcoming the well documented technical difficulties [7] (in identifying the fenestration site, and in providing the mechanical support during the fenestration) that had slowed the adoption of the antegrade approach for the endograft intraoperative modification.

The proposed technology aims at expanding the indications for EVAR, to patients with unfavorable anatomy (e.g. short-necked aneurysms), even in the setting of ruptured or symptomatic AAAs which, nowadays, are treated with an open surgical approach, due to the high lead-time of fenestrated endograft. Thus, the potential clinical impact is significant, since the development of a computer-assisted method and tools for the execution of antegrade ISF can allow a minimally-invasive endovascular approach to be performed in patients that are nowadays considered ineligible for an EVAR procedure. Moreover, the proposed approach paves the way to use ISF as a bailout technique for rescuing branched aortic arteries, e.g. renal arteries, after inadvertent coverage during EVAR, avoiding permanent failure of vital organs.

A major limitation of the present technology is that the navigation software is based on a static representation of the anatomy. For this reason, planned future works include: in vivo animal studies to verify the targeting accuracy of the system in presence of physiological movements due to cardiac cycle/respiration and deformation due to endograft delivery; the development of strategies to compensate for these movements/deformations based on a real time X-ray free tracking of target aorta branches. In addition, the optimization of mechanical properties of the sensorized laser guidewire is necessary to facilitate its advancement within the fenestration to make the ISF technique more repeatable and safer. For the in vivo testing, particular attention will be paid to user performance in terms of the number of attempts at ISF and the completion time.
